# The impacts of muscle-specific force-velocity properties on predictions of mouse muscle function during locomotion

**DOI:** 10.3389/fbioe.2024.1436004

**Published:** 2024-07-23

**Authors:** James P. Charles, Roger W. P. Kissane, Graham N. Askew

**Affiliations:** ^1^ Department of Musculoskeletal and Ageing Science, University of Liverpool, The William Henry Duncan Building, Liverpool, United Kingdom; ^2^ School of Biomedical Sciences, University of Leeds, Leeds, United Kingdom

**Keywords:** force velocity, muscle mechanics, musculoskeletal model, lengthening, muscle work

## Abstract

**Introduction:** The accuracy of musculoskeletal models and simulations as methods for predicting muscle functional outputs is always improving. However, even the most complex models contain various assumptions and simplifications in how muscle force generation is simulated. One common example is the application of a generalised (“generic”) force-velocity relationship, derived from a limited data set to each muscle within a model, uniformly across all muscles irrespective of whether those muscles have “fast” or “slow” contractile properties.

**Methods:** Using a previously built and validated musculoskeletal model and simulation of trotting in the mouse hindlimb, this work examines the predicted functional impact of applying muscle-specific force-velocity properties to typically fast (extensor digitorum longus; EDL) and slow-contracting (soleus; SOL) muscles.

**Results:** Using “real” data led to EDL producing more positive work and acting significantly more spring-like, and soleus producing more negative work and acting more brake-like in function compared to muscles modelled using “generic” force-velocity data. Extrapolating these force-velocity properties to other muscles considered “fast” or “slow” also substantially impacted their predicted function. Importantly, this also further impacted EDL and SOL function beyond that seen when changing only their properties alone, to a point where they show an improved match to *ex vivo* experimental data.

**Discussion:** These data suggest that further improvements to how musculoskeletal models and simulations predict muscle function should include the use of different values defining their force-velocity relationship depending on their fibre-type composition.

## Introduction

Computational musculotendon models, such as the commonly used Hill-type model (Hill, 1938), are widely used methods to predict muscle functional outputs (Zajac, 1989). These models are often incorporated into biomechanical models of a vertebrate musculoskeletal system. They provide invaluable insights into healthy and pathological muscle dynamics where it is typically not possible to quantify performance using traditional experimental methods (Seth et al., 2018; Rajagopal et al., 2016; Hutchinson et al., 2015; Arnold et al., 2010; Falisse et al., 2019; Charles et al., 2016b; O’Neill et al., 2013; Steele et al., 2012). However, it is accepted that such models are merely approximations of *in situ* muscle contractile behaviour and carry several inherent assumptions including, among others, the use of standardised “generic” values to characterise their force-generating properties (Ward et al., 2009; Rajagopal et al., 2016).

The impacts of these assumptions have been explored in an effort to improve how models predict individual muscle function, with improved predictions of muscle output generated by tuning muscle parameters to match the dimensions of individuals (Modenese et al., 2016) or the inclusion of subject-specific muscle architecture data, such as fibre lengths and physiological cross-sectional area (Charles et al., 2020). However, little attention has been given towards inter-muscle variations in muscle fibre phenotype, and thus differences in the force-velocity relationship, and its impact on model outputs (Millard et al., 2013).

The force-velocity relationship describes the complex force-generating capacity of a muscle as it undergoes active shortening or lengthening. Muscles that undergo active shortening (i.e., concentric contraction) present with a reduction in force-generating capacity as shortening velocity increases (Hill, 1938). While muscles that undergo active muscle lengthening (i.e., eccentric contractions) display an enhancement in force-generating capacity as lengthening velocity increases (Katz, 1939), which is greater than the maximum isometric force capability of the muscle (Joyce et al., 1969). It has been comprehensively established that muscles with differing fibre phenotype present with distinctly different shortening force-velocity relationships (Askew and Marsh, 1997). For instance, slower muscles typically present with force-velocity relationships with greater curvature and lower maximum speeds of muscle shortening when compared to faster phenotype muscles (Askew and Marsh, 1997). Contrastingly, the influence of muscle fibre phenotype on the lengthening force-velocity relationship has been comparably less well investigated. There is however a growing body of evidence that suggests that the rate of force development during eccentric muscle contractions (Linari et al., 2004) and the plateau height of the eccentric force-velocity relationship differs between slower and faster muscles (Kissane and Askew, 2024). It is clear that across the dynamic force-velocity relationship, muscle fibre phenotype appears to have a significant bearing on force estimates for a given velocity of lengthening and shortening. However, due to a lack of relevant data needed to scale individual muscle force-velocity curves and a common need to reduce model complexity, these phenotypic variations are rarely accounted for within computational muscle models. Instead, this relationship, in models of species ranging from humans (Rajagopal et al., 2016) to mice (Charles et al., 2016b), has traditionally been informed in all muscles by a hybrid of “generic vertebrate” force-velocity data (Joyce et al., 1969; Mashima, 1984; Millard et al., 2013), which is unlikely to represent either phenotypically fast or slow muscles in certain species. Therefore, the exact impact that the use of these data has on musculoskeletal model estimates warrants in-depth investigation, in order to improve how the functions of muscles of varying morphologies and fibres phenotypes are predicted *via* computational models.

Here, the aim was to incorporate force-velocity data from 2 mouse muscles, the extensor digitorum longus muscle (EDL; a typical fast muscle) and the soleus muscle (SOL; a typical slow muscle) into a previously developed musculoskeletal model of the mouse hindlimb and pelvis, to explore the implications of force-velocity variations in muscles of differing fibre phenotype on predictions of muscle force, work and function during trotting locomotion.

## Materials and methods

### Musculoskeletal models and simulations

A previously published musculoskeletal model of the mouse hindlimb and pelvis (Charles et al., 2016b) was used as the basis of the model used here. That model was modified to include the opposite hindlimb as well as whole-body cranial-caudal and dorsal-ventral translations, the “Millardequilibrium 2012” musculotendon model to predict activation dynamics of each musculotendon unit (Millard et al., 2013), and was updated to be compatible with the latest version of Opensim [v4.5 (Seth et al., 2018)]. Opensim’s Scale tool was then used to scale the size and mass and inertial properties of this model to match the body mass of eleven male C57BL/6 mice (Body mass- 25.3 ± 2.1 g, Age- 10–12 weeks old; Supplementary Tables S1, S2; see “*ex vivo* validation” for further use of these mice). The optimal fibre length (L_f_’) and maximum isometric force (F_max_) values for EDL and SOL were amended in each model based on muscle architecture data measured from the same mice through dissections (Supplementary Table S2), where F_max_ was calculated from physiological cross-sectional area as per Charles et al. (2016a). To investigate the impacts of applying muscle-specific force-velocity properties to each mouse model, two model variants of each individual mouse were created.

**FIGURE 1:**
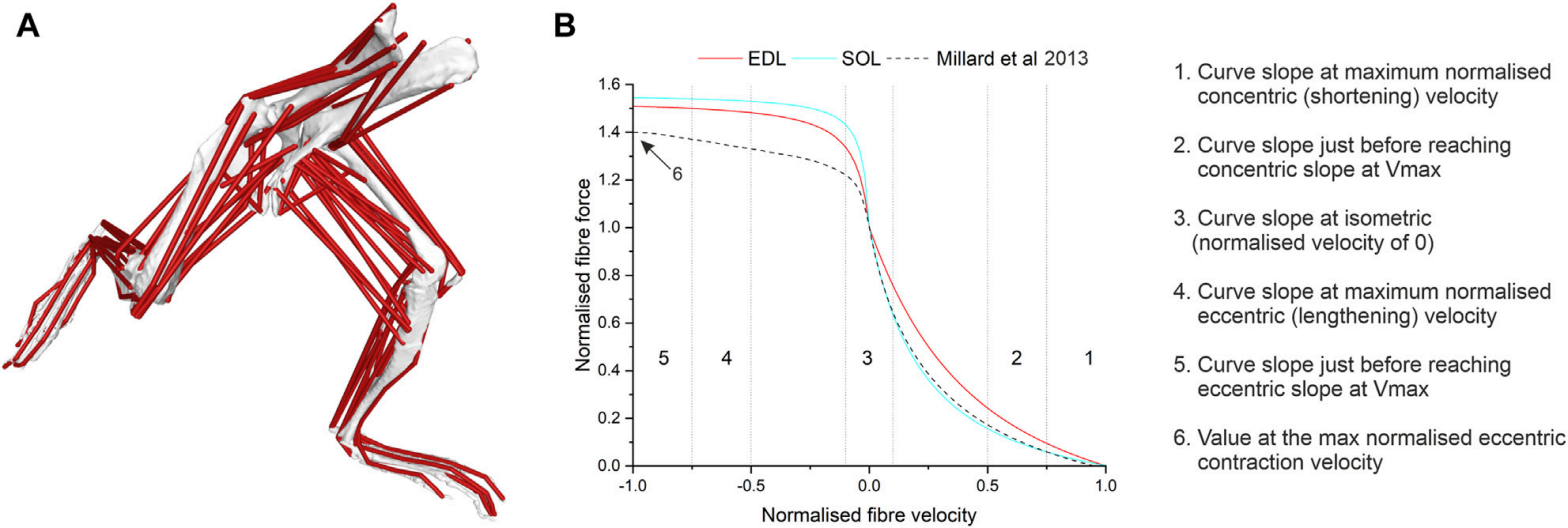
Within a musculoskeletal model of the mouse hindlimb and pelvis **(A)**, several factors of the force-velocity curve **(B)** in the extensor digitorum longus (EDL) and soleus (SOL) muscle were amended from the generic curve (Millard et al., 2013) to new values based on muscle-specific data (Kissane et al., 2024).

- Generic-default, or generic, force-velocity properties for each muscle as described by Millard (Millard et al., 2013).
- Real- Force-velocity data derived from Kissane and Askew (2024) used to modify force-velocity curves in the EDL and soleus (see Figure 1; Supplementary Figure S1; Table 1 for the specific properties amended in each model)

**TABLE 1:** Force-velocity properties of the Millard muscle model amended here with “fast” and “slow” phenotype-specific data, from Kissane and Askew (2024).

	Generic	EDL (“fast”)	SOL (“slow”)
Maximum contraction velocity (L_f_’ s^-1^)	10	14.13	7.01
Activation time constant	0.01	0.008	0.017
Deactivation time constant	0.04	0.032	0.063
Curve slope at maximum normalised concentric (shortening) velocity	0.125	0.382	0.23
Curve slope just before reaching concentric slope at maximum normalised concentric (shortening) velocity	0.25	0.585	0.381
Curve slope at isometric (normalised velocity of 0)	5	2.87	3.97
Curve slope at maximum normalised eccentric (lengthening) velocity	0.1	0.038	0.021
Curve slope just before reaching eccentric slope at maximum normalised eccentric (lengthening) velocity	0.15	0.067	0.038
Force value at the maximum normalised eccentric contraction velocity	1.4	1.51	1.54

To investigate the impacts of applying these experimentally derived force-velocity properties to other muscles of similar phenotypes, an “extrapolated” model variant of each mouse was created. Here, each muscle in the model was classed as either “fast”, “slow” or “intermediate” based on their relative proportions of Type IIb fibres, as reported by Burkholder et al. (1994) (Supplementary Figure S2). The EDL-derived “fast” force-velocity properties were applied to each “fast” muscle, while the SOL-derived “slow” force-velocity properties were applied to each “slow” muscle. Muscles with relatively equal slow and fast fibre proportions were classified as “intermediate” and were left unchanged in this model.

In each of these model variants, motion data and external forces (i.e., ground reaction forces) from Charles et al. (2018) were used to generate simulations of a single gait cycle of trotting locomotion. In this previous work, hindlimb joint kinematics were measured from high-speed videos of 5 mice trotting along a clear walkway (average velocity- 0.59 ms^-1^), with an embedded six-axis (3-force axes, 3-moment axes) custom-built strain gauge-based acrylic force plate (7.5 cm × 7.5 cm, recording rate 2500 Hz) measuring ground reaction forces. The gait cycle (toe-off to toe-off) which closely matched the average from the 5 mice in terms of hindlimb joint angles was chosen to produce the trotting simulation in each mouse model, with the measured ground reaction forces scaled to each mouse’s body weight. With these data, the Static Optimisation tool within OpenSim (with the objective function to minimise squared muscle activations) was used to predict the muscle forces and activations necessary to satisfy the external forces during this motion in each model variant.

From the outputs of each of these simulations, individual muscle instantaneous power was calculated by multiplying instantaneous force by instantaneous contraction velocity, with positive and negative work calculated by integrating these power curves during the shortening and lengthening phases, respectively. Individual muscle function was then quantified through the calculation of dimensionless functional indices (Lai et al., 2019), which, based on the timing of force and work production throughout a gait cycle (see Supplementary Material), class muscles as functioning as either motors (predominantly positive work generation), brakes (predominantly negative work generation), springs (equal negative and positive work generation) or struts (little work generation), with each function expressed as a percentage (see Supplementary Material for more information on the calculation of these indices). A muscle’s “primary” function was assigned depending on its largest index. All muscle data presented are for muscles from the right hindlimb.

### *Ex vivo* validation

All *ex vivo* experimental procedures described here were performed in accordance with the United Kingdom Animal Scientific Procedures Act (1986) and approved by the University of Leeds Animal Welfare and Ethical Review Committee. This work conforms to the ethical requirements outlined by the journal and is presented in accordance with guidelines for animal work (Percie du Sert et al., 2020). Eleven male C57BL/6 mice (Body mass- 25.3 ± 2.1 g, Age- 10–12 weeks old; Supplementary Table S2) were used in this aspect of the study. Animals were housed under a 12-hour light:dark cycle at 21°C and had *ad libitum* access to food and water.

An *ex vivo* work loop approach was used to validate the muscle forces, power and work predicted by each musculoskeletal model. Here, the eleven mice described above were culled using approved schedule 1 methods, after which both hindlimbs were transferred to chilled (4°C), oxygenated (95% O_2_, 5% CO_2_) Krebs-Henseleit solution [117 NaCl, 4.7 KCl, 2.5 CaCl_2_, 1.2 MgSO_4_, 24.8 NaHCO_3_, 1.2 KH_2_PO_4_ and 11.1 glucose; concentrations in mmol L^-1^] (Burton, 1975). The SOL and EDL were dissected free and aluminium foil clips were attached to small portions of the proximal and distal tendons, leaving no free tendon in series between the clip and the muscle belly (Askew and Marsh, 1997). The muscles were suspended vertically in a Perspex flow-through chamber filled with circulating, oxygenated Krebs–Henseleit solution at 37°C ± 0.5°C. Muscles were attached to an ergometer (series 300B-LR; Aurora Scientific Inc., London, Ontario, Canada) *via* a lightweight stainless-steel rod; the position of the ergometer and therefore the length of the muscle could be controlled using a digital height gauge (Mitutoyo Corporation, Kanagawa, Japan). Muscles were left for 30 min to thermoequilibrate. Parallel platinum electrodes were placed inside the chamber on either side of and parallel to the muscle.

All muscles were subjected to a series of supramaximal isometric twitches (0.2 ms pulse width) and incrementally lengthened to find the optimal length for maximum twitch force generation, which was used as the length about which the muscle was oscillated throughout the experiments. Using a modified work loop approach the SOL and EDL were subjected to strain trajectories and activation patterns derived from the Extrapolated musculoskeletal mouse model (Supplementary Figure S3). The strain trajectories were imposed on the muscles using the ergometer and muscle activation patterns were delivered *via* a custom-built stimulator (modified 701C stimulator, Aurora Scientific, Aurora, Ontario, Canada) (Bukovec et al., 2020) with both devices being controlled using custom-written protocols (Dynamic Muscle Control software, Aurora Scientific, Aurora, Ontario, Canada). Muscle activation levels (Supplementary Figure S2A) were calculated using a current-recruitment curve, where the threshold currents required to maximally activate (upper threshold) and to minimally activate (lower threshold) the muscle were determined. Subsequently, the activation curves were calculated for each individual muscle for the muscle work loop experiments. As with the model outputs, instantaneous power was calculated by multiplying instantaneous force by instantaneous contraction velocity, with positive and negative work calculated by integrating these power curves during the shortening and lengthening phases, respectively. Net power was also calculated as the average of instantaneous power over the cycle.

The experimentally derived force, power and work outputs of the EDL and SOL muscle bellies were used to quantify *ex vivo* functions using the same functional indices calculations used above. The fibre (i.e., non-tendinous portions of the whole musculotendon unit models, therefore analogous to an *ex vivo* muscle minus an external tendon) power outputs and functions of these same muscles predicted by the “extrapolated” models were compared to these *ex vivo* outputs. As Static Optimisation does not strictly predict fibre force in isolation from whole musculotendon unit force, these “fibres” were modelled by creating analogous actuators for EDL and SOL of the same length as their optimal fibre length (with the same proximal origin point), with their tendon slack length reduced to a value which produced the same normalised fibre lengths as their whole muscle-tendon unit counterparts throughout the trotting gait cycle.

A summary of this experimental workflow is shown in Supplementary Figure S4.

### Statistical analyses

One-way analyses of variance (ANOVA) were used to test for differences in muscle work outputs functional indices between the Generic and Real models. The root mean squared (RMS) errors of the power outputs of the EDL and SOL fibres were calculated for the Generic and Extrapolated models relative to the *ex vivo* data. 1D statistical parametric mapping [SPM; (Pataky, 2010)] was used to test for significant differences in EDL and SOL force and power outputs between the Generic and Real models throughout the gait cycle and was performed in MATLAB (v. 2023b; MathWorks, Natick, MA, United States). The SPM and RMSE calculations were carried out in MATLAB, while the ANOVAs were performed using OriginLab software (OriginLab Corporation, Northampton, MA, United States).

## Results

### Generic vs. real

Table 1 shows the newly defined force-velocity relationship characteristics (“Real”) derived from Kissane and Askew (2024) for both fast and slow muscles, compared to the generic properties often used to define this relationship in computational musculotendon models.

**FIGURE 2:**
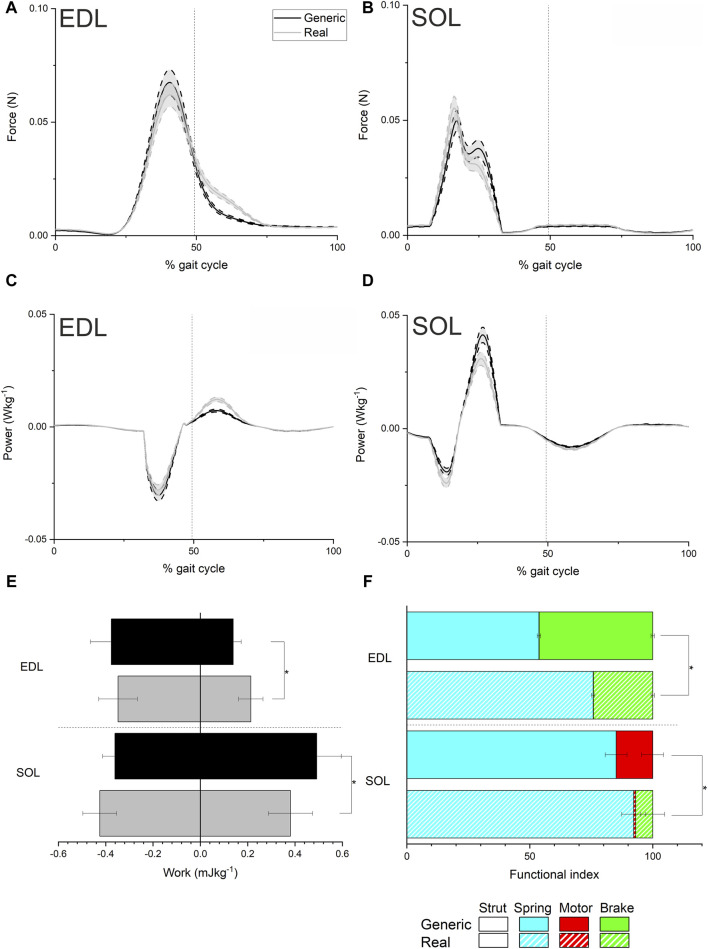
Mean ± standard error musculotendon unit force **(A and B)**, instantaneous power **(C and D)**, work **(E)** and functional indices **(F)** from the simulations of trotting locomotion in each mouse model using generic and real force-velocity properties in the extensor digitorum longus (EDL) and Soleus (SOL). Both work and inferred function were significantly impacted in EDL and SOL by using experimentally derived force-velocity properties. * indicates a statistically significant difference (*p* < 0.05).

The simulations informed by the generic data (“Generic” simulations), predicted peak forces of 0.067 N in EDL and 0.049 N in SOL, when averaged over each mouse, while the Real simulations predicted average peak forces of 0.062 N in EDL and 0.055 N in SOL (Figures 2A, B). In EDL there was a decrease in peak negative power during the stance phase (first half of the gait cycle) in the Real models compared to the Generic (from −0.030 W kg^-1^ to −0.028 W kg^-1^), but an increase in peak instantaneous positive power during the swing phase (from 0.007 W kg^-1^ to 0.012 W kg^-1^; Figure 2C). The opposite occurred in SOL, with an increase in peak negative instantaneous power from −0.019 W kg^-1^ to −0.024 W kg^-1^ in the Real model, and a decrease in peak positive power from 0.040 W kg^-1^ to 0.031 W kg^-1^, both during the stance phase (Figure 2D). These differences in peak force and peak instantaneous power values were not statistically significant (Supplementary Figure S5), with SPM only finding slight statistically significant differences after (∼60–65% gait cycle) periods of activation and force generation in EDL. These changes in instantaneous power did however translate to significant differences in mechanical work generation (Figure 2E), with a significant increase in positive work in EDL (from 0.13 ± 0.03 J kg^-1^ to 0.21 ± 0.05 J kg^-1^, *p* =< 0.01), and a significant decrease in positive work in SOL (from 0.49 ± 0.09 J kg^-1^ to 0.38 ± 0.10 J kg^-1^, *p* = 0.04). There were also slight decreases in negative work in EDL (−0.37 ± 0.08 J kg^-1^ to −0.34 ± 0.08 J kg^-1^) and increases in negative work in SOL (from −0.42 ± 0.07 J kg^-1^ to −0.36 ± 0.05 J kg^-1^), although these differences were not statistically significant (*p* = 0.48 and *p* = 0.06 respectively).

Functional indices significantly changed in both EDL and SOL between the Generic and Real models (Figure 2F), with a significant shift towards a spring-like function in EDL (from 54% ± 0.69% to 76% ± 0.66%; *p* = < 0.001) and a significantly higher braking function in SOL (from 0.07% ± 0.005% to 6.97% ± 4.87%; *p* = < 0.001).

### Extrapolated model

**FIGURE 3:**
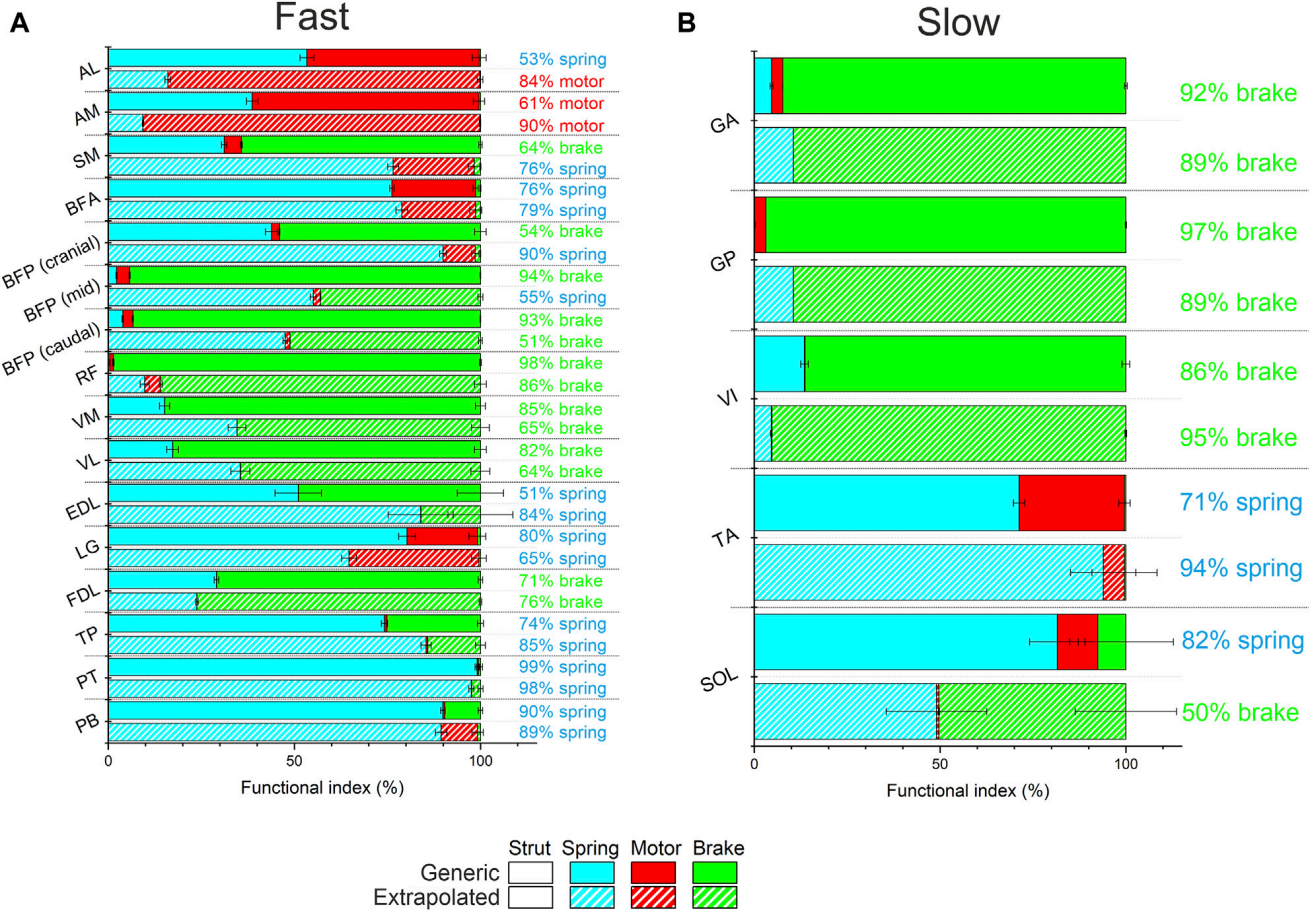
Functional indices of each fast **(A)** and slow **(B)** musculotendon unit with both generic force-velocity data and real force-velocity data extrapolated from extensor digitorum longus and soleus to these muscles. Muscles were classed as fast or slow based on their fibre type profiles (see Supplementary Figure S1). The fast muscles within the Extrapolated models showed more widespread and larger differences in function compared to the Default models, relative to the slow muscles where soleus (SOL) showed the largest functional discrepancies. Muscle definitions: AL-adductor longus, AM-adductor magnus, SM-semimembranosus, BFA-biceps femoris (anterior), BFP- Biceps femoris (posterior), RF- rectus femoris, VM-vastus medialis, VL-vastus lateralis, EDL-extensor digitorum longus, LG-lateral gastrocnemius, FDL-flexor digitorum longus, TP- tibialis posterior, PT-peroneus tertius, PB- peroneus brevis, GA-gracilis anterior, GP- gracilis posterior, VI- vastus intermedius, TA-tibialis anterior, SOL-soleus.

The musculotendon functions predicted by the Extrapolated models differed substantially in numerous instances relative to the Generic model (Figure 3), particularly in the “fast” muscles (Figure 3A). For instance, the adductor longus (AL) and adductor magnus (AM) muscles became more motor-like in function relative to the equivalent Generic model (AL- 53% spring to 84% motor; AM- 61%–90% motor), while semimembranosus (SM), biceps femoris posterior (BFP) cranial and mid muscles all changed from functioning as brakes to springs (SM- 64% brake to 76% spring; BFP cranial- 54% brake to 90% spring; BFP mid- 94% brake to 55% spring). The functional changes in the more distal “fast” muscles were less substantial than the proximal muscles, however there were large changes in the spring index of EDL (from 51% to 84%) and LG (from 80% to 65%). There were also less notable functional changes in most of the “slow” muscles within the Extrapolated models (Figure 3B), with SOL showing the largest change with a change from a spring function (82%) to a predominantly braking function (50%), and TA showing an increase in spring-like function (from 71% to 94%).

### *Ex vivo* validation

**FIGURE 4:**
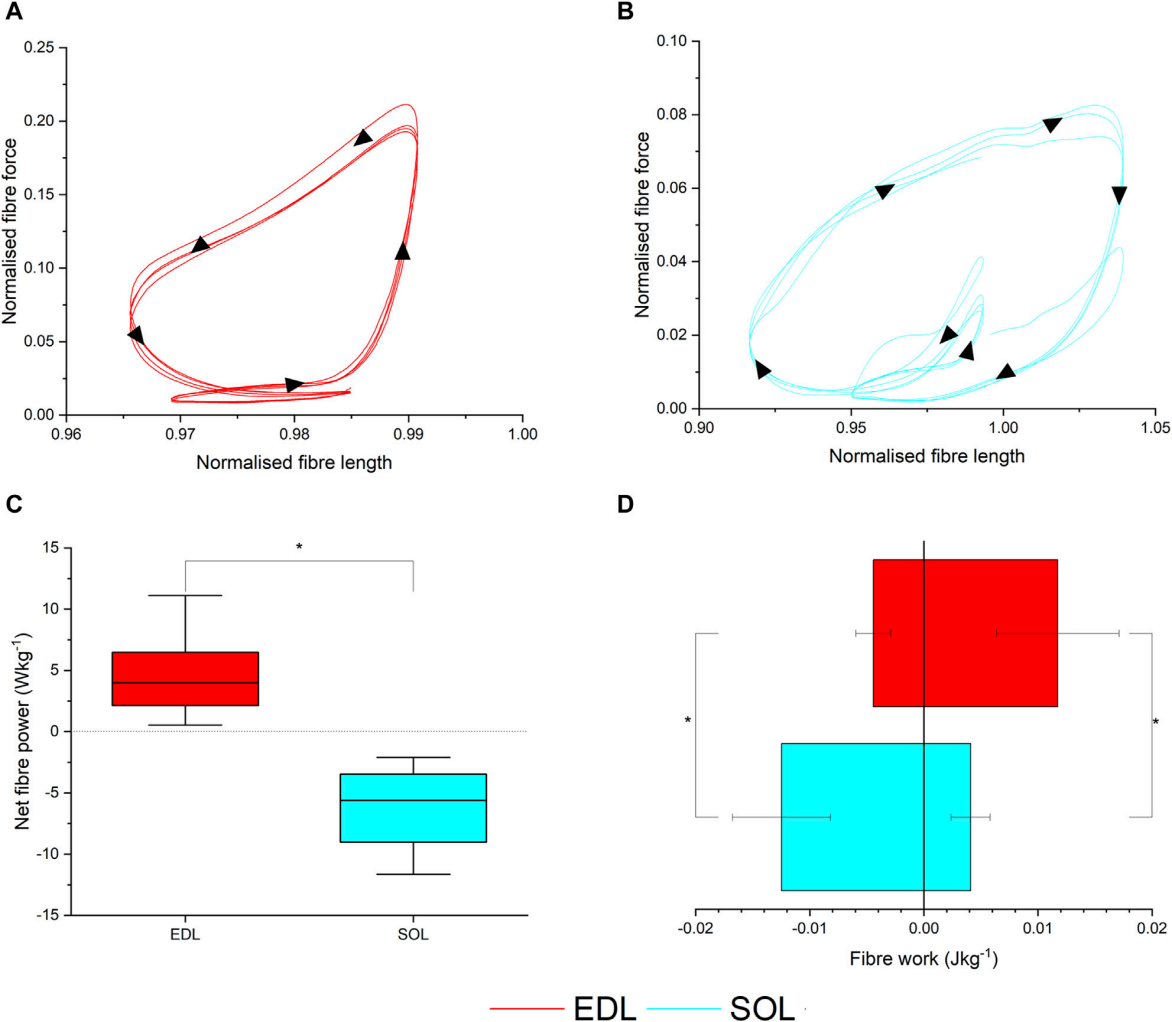
*Ex vivo* muscle mechanical properties. Muscle work loops for the extensor digitorum longus (EDL) **(A)** and soleus (SOL) muscles **(B)**, and corresponding net fibre power **(C)** and positive and negative work per cycle **(D)**.

Using the musculoskeletal model-derived fibre length trajectories and activation patterns (Figures 4A, B; Supplementary Figure S3), the *ex vivo* work loop protocol showed that during gait, EDL produced significantly greater net fibre power (4.50 ± 3.19 W kg^-1^) compared to SOL (−7.60 ± 6.68 W kg^-1^, *p* =< 0.001; Figure 4C), as a consequence of generating significantly less negative work (EDL = −0.004 ± 0.001 J kg^-1^; SOL = −0.012 ± 0.004 J kg^-1^, *p* =< 0.001; Figure 4D) and more positive work (EDL = 0.011 ± 0.005 J kg^-1^; SOL = 0.004 ± 0.004 J kg^-1^, *p* =< 0.001; Figure 4D).

**FIGURE 5:**
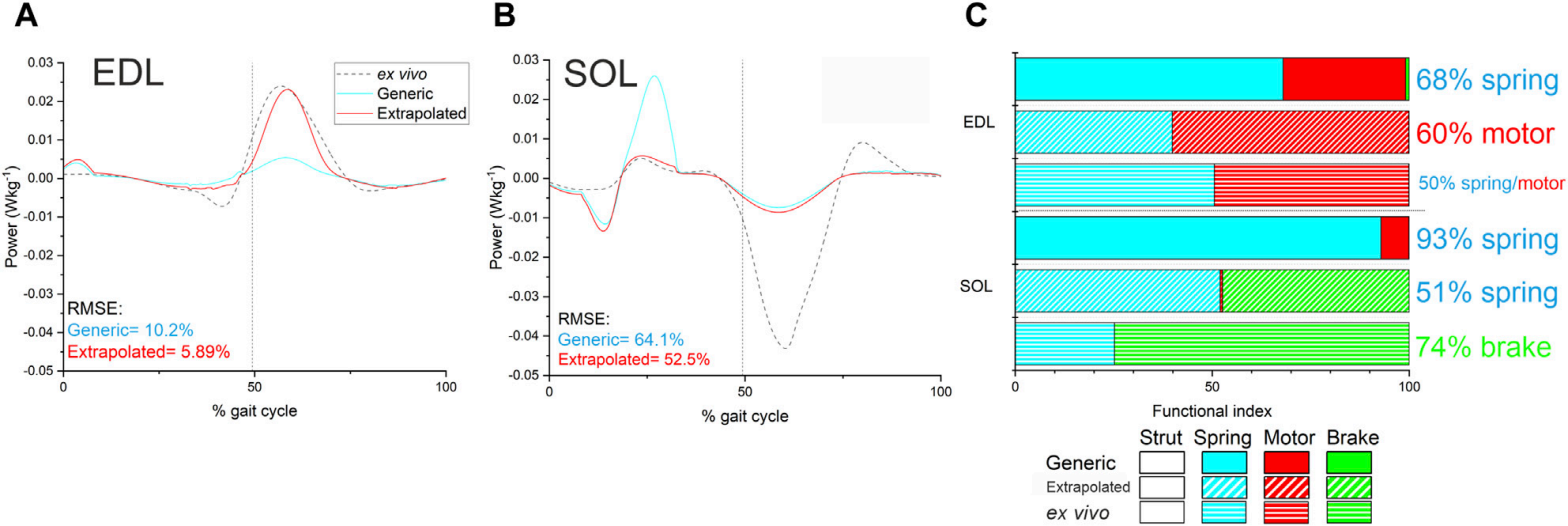
Instantaneous fibre power **(A and B)** and function **(C)** in EDL and SOL as predicted by the Generic and Extrapolated models, as well as those experimentally determined through *ex vivo* methods. RMS errors in fibre instantaneous power were lower in both muscles in the Extrapolated model, and there were less discrepancies in inferred function compared to the Generic model.

The predicted instantaneous fibre power generated by the EDL in the Extrapolated models showed a closer match to the *ex vivo* data than the Generic model of the same mouse (RMSE- Generic = 10.2%, Real = 5.89%; Figure 5A). The RMS errors in SOL instantaneous power were much larger in both the Extrapolated and Generic models (Figure 5B) due to a large degree of negative work during the swing phase measured *ex vivo*, which was not captured in the model predictions, although errors were lower in the Extrapolated model due to a close match during the stance phase (Generic = 64.1%, Real = 52.5%). As a result, the fibre functions in the Extrapolated models showed closer matches to the *ex vivo* data in both EDL (Generic = 68% spring, 31% motor; Extrapolated = 40% spring, 60% motor; *ex vivo* = 51% spring, 49% motor) and SOL (Generic = 92% spring, 7% motor; Extrapolated = 52% spring, 47% brake; *ex vivo* = 25% spring, 75% brake; Figure 5C).

## Discussion

In the context of computational musculotendon models, the most in-depth representation of the force-velocity relationship (Millard et al., 2013) was informed by limited data sets obtained from cat (Joyce et al., 1969) and frog (Mashima, 1984) muscles, thus creating a “generic vertebrate” data set which is traditionally applied to all muscles. By applying newly derived muscle-specific force-velocity curves into these established models of musculotendon force generation via a musculoskeletal model and simulation of trotting locomotion in the mouse hindlimb, this study (see also Supplementary Material) found a high level of sensitivity of muscle functional outputs to changes in this relationship. Specifically, significant differences in both muscle net work and overall function of the EDL and SOL were found in response to the application of these muscle-specific data rather than “generic vertebrate” force-velocity curves. Furthermore, applying these values to multiple muscles produced model outputs with closer matches to *in vivo* muscle physiology and thus may be needed to accurately infer vertebrate muscle function using computational models.

While the majority of musculoskeletal modelling and simulation research has been performed on the human musculoskeletal system, thus providing the most in-depth knowledge of individual muscle functions during dynamic movements, a model of the mouse hindlimb and pelvis was chosen as the platform for this work for two reasons: 1) mice are commonly used models for several human neuromuscular diseases (Willmann et al., 2009; Henriques et al., 2010; Malerba et al., 2011; Sharp et al., 2011; Partridge, 2013), meaning that an increase in the accuracy of muscle functional predictions could be crucial in informing patient treatments, and 2) mice hindlimb muscles, particularly EDL and SOL, are often used in *ex vivo* muscle physiology preparations due to their morphology and distinct muscle fibre phenotypes (EDL- 100% Type II “fast” fibres, SOL- 58% Type I “slow” fibres). These muscles are therefore an ideal platform on which to generate a more accurate picture of the mammalian force-velocity relationship.

It has long been established that concentric force-velocity properties differ between of fast- and slow-phenotype muscles (Luff, 1981; Askew and Marsh, 1997), yet conflicting data exist on the eccentric force-velocity properties between fast and slow muscles. Some studies (Rijkelijkhuizen et al., 2003) have suggested no difference exists among phenotypically different muscles, while others have reported subtle velocity-specific differences (Stienen et al., 1992; Ramsey et al., 2010). It has been recently shown that the “fast” EDL and “slow” SOL have significantly different eccentric and concentric force-velocity profiles (Kissane and Askew, 2024), with the SOL attaining a greater plateau height and more curved force-velocity profile compared to EDL during the eccentric force-velocity relationship. These observations are similar to those seen in phenotypically distinct isolated muscle fibres from humans (Linari et al., 2004). The mechanisms behind any difference in eccentric contractile properties, are however, still debated (Alcazar et al., 2019). The difficulty in ascertaining a singular mechanism for fibre type differences in the eccentric force-velocity relationship is in part because of the dynamic bi-phasic force response to active muscle lengthening (Katz, 1939). Muscles undergoing eccentric activation comprise an initial (phase-1) rapid increase in force, which is thought to be in response to elevated strain of attached cross-bridges. This is followed by the detachment of myosin heads, which leads to the transition into a shallower phase-2 force response (Katz, 1939). This phase-2 force response is thought to be linked to an increased strain of non-cross-bridge parallel elastic elements (e.g., titin) (Tomalka et al., 2017; Tomalka et al., 2020; Weidner et al., 2022; Tomalka, 2023). The phase-2 portion is the region that is sampled (for the P/P_0_ at any given velocity of lengthening) to derive the classical force-velocity relationship (Figure 1B) and this phase is dependent on normal titin function (Kissane and Askew, 2024). Further, it is known that phenotypically distinct muscles may be comprised of different isoforms of titin (Hettige et al., 2020; Hettige et al., 2022), meaning differences that exist in the hyperbolic force-velocity relationship could be attributed to differences in titin. To date, no attempt has been made to incorporate the dynamic velocity-dependent phase-1 and phase-2 behaviour into computational muscle modelling, meaning that such models are reliant upon the classical double hyperbolic concentric and eccentric force-velocity relationship in order to predict muscle dynamics and functions.

The force-velocity properties extracted from both the fast phenotypic and slow phenotypic concentric and eccentric contraction curves outlined by Kissane and Askew (2024) produced values that were, in some cases, substantially different to those from the current default force–velocity relationship (Millard et al., 2013). Perhaps most importantly, the “generic vertebrate” data has a normalised force value at the maximum normalised eccentric contraction velocity of 1.4, which underestimates the height of this plateau in “fast” muscles by 7.2% and “slow” muscles by 9%. Critically, this “generic vertebrate” value has been derived from muscles of cats at body temperature and frog muscles at 10°C, which when compared to a mammalian muscle only data set (Kissane and Askew, 2024), results in a normalised force value at the maximum normalised eccentric contraction velocity of 1.65 (18% higher). While these may be considered small differences, the model outputs were most sensitive to this parameter (see ESM, Supplementary Tables S3 and S4) and this sensitivity translated to larger changes in outputs from the EDL and SOL muscles, with a 61% increase in positive work and a 41% increase in the spring-like function in the EDL and a 22% decrease in positive work output from SOL.

The calculation of dimensionless muscle functional indices (Charles et al., 2018) allows for an investigation of individual mouse muscle function beyond what has been previously inferred from muscle anatomy alone (Burkholder et al., 1994; Charles et al., 2016a) or previous simulations (Charles et al., 2018). While classifying a complex and heterogeneous structure like skeletal muscle as acting primarily as one of only four functional roles could be seen as crude, with a further possibility of misclassification due to the cyclic nature of trotting accentuating spring-indices (Lai et al., 2019), their use here has crucially allowed for the sensitivity in model outputs to the force-velocity relationship to be placed into an important functional context. With these indices, it was found that despite large differences in some areas of the EDL and SOL force-velocity curves from a previously used generic curve (Figure 1B), the overall functional inferences of these muscles remained similar between the Generic and Real models (EDL-spring/brake, SOL-spring) when only the properties of these muscles were changed (Figure 2F). This is not entirely unexpected, given the large non-contractile tendinous proportion of EDL (57% - Charles et al. (2016a)) and the relatively low activations and force productions of both muscles, thus low functional importance, during these trotting simulations (Charles et al., 2016b). However, extrapolating these phenotype-specific properties other muscles of similar fibre type compositions but differing morphologies (i.e., larger proportion of muscle contractile fibres) and larger functional importance during dynamic movements such as trotting revealed larger changes in inferred function (Figure 3). This “extrapolated” model predicted total changes in inferred function in several muscles including adductor longus (AL-from spring to motor), semimembranosus (SM-from brake to spring) and biceps femoris posterior (BFP- from brake to spring), and in general an increase in the motor function (i.e., positive work generation) of the powerful proximal hindlimb muscles (as well as a larger relative metabolic output; Supplementary Figure S4). While trotting is the most common movement used by freely moving mice (Mendes et al., 2015), different results will likely be generated by simulating different movements. In slower movements requiring more fatigue resistance or stability, such as walking or incline locomotion (Sasaki and Neptune, 2006; Franz and Kram, 2012), larger changes in the Extrapolated models may be seen in the muscles with larger proportions of slower fibre types, which would be more active and functionally important during these gaits. Investigating these differences could be an interesting area for future work, particularly when attempting to critique the relevance of mice as models for various human neuromuscular conditions, such as Duchenne Muscular Dystrophy (Willmann et al., 2009; Partridge, 2013; Aartsma-Rus and van Putten, 2014) or sarcopenia (Xie et al., 2021).

It may also be seen as a gross simplification to represent certain muscles with only one set of force-velocity characteristics. For instance, it is known that, across mammals, muscles such as the tibialis anterior and extensor digitorum longus are particularly heterogeneous in their fibre type composition, leading to significant inter-compartment differences in power generation (Kissane et al., 2018). It is therefore possible that a more accurate method of incorporating muscle-specific fibre-type properties into musculoskeletal models would be to represent demonstrably heterogeneous muscles such as these with multiple musculotendon unit actuators with distinct force-velocity characteristics, which is worthy of further in-depth analysis.

Ultimately, the lower RMS errors in EDL and SOL muscle power and more similar muscle functions to those measured from the *ex vivo* preparation, highlight the increased accuracy of simulations including muscle-specific force-velocity properties compared to those using generic properties. There were, however, discrepancies in predictions of instantaneous negative power in SOL within the Extrapolated model relative to the *ex vivo* data, especially during the swing phase. It is possible these arose from the high passive forces generated by SOL *ex vivo* due to its force-length relationship (Kissane and Askew, 2024), and the use of Static Optimisation to predict muscle activations and forces in the model, which ignores passive fibre force generation (Rankin et al., 2016). It is therefore possible that with further improvements to the model (i.e., better representations of body segments other than the hindlimb) and its input data used to generate simulations (i.e., more accurate kinematics or forces applied to the forelimbs), more sophisticated techniques to predict muscle dynamics which do not ignore these passive forces, such as Computed Muscle Control (CMC) (Thelen et al., 2003) or complex predictive optimal control simulations (Falisse et al., 2019; Bishop et al., 2021), will generate predictions of muscle function closer to *ex vivo* data compared to those presented here. It is also likely that these predictive methods will produce more realistic limb kinematics when supplemented with muscle or phenotype-specific force-velocity properties, which would benefit further work into predictions of functional improvements in response to neuromuscular disease treatments and interventions using this model. Despite these differences, the improvements in model output accuracy presented here highlight the potential need to change the properties of multiple muscles to accurately predict single muscle function. In fact, it is possible that using these different methods of predicting muscle functions, which also incorporate muscle excitation-activation dynamics and more complex calculations of tendon dynamics, will even further highlight the benefits of including muscle or phenotype-specific force-velocity characteristics within musculoskeletal models.

Overall, this work has highlighted the high sensitivity of muscle force, power and work outputs from computational models and simulations to changes in the force-velocity relationship. It also heavily supports the inclusion of muscle-specific, or at least phenotype-specific, force-velocity properties in muscle models of force generation when inferring individual muscle function. Of course, this work did not consider all aspects of muscle physiology, with the functional impacts of changes to the active and passive force-length relationships of the fibres or tendons out of the scope of this study. However, it is possible that applying muscle-specific force-length data to muscle models, if available, may further improve the accuracy of their output beyond what has been shown here. Further work will also seek to investigate the impacts of applying these force-velocity properties to models of other vertebrates, such as humans, where more accurate predictions of individual muscle function could have significant impacts in clinical, sports science or robotics research (Li et al., 2018; Ceccarelli et al., 2020; Ficht and Behnke, 2021).

## Data Availability

The raw data supporting the conclusions of this article will be made available by the authors, without undue reservation. The musculoskeletal model of the mouse hindlimb is available to download here: https://simtk.org/projects/mousehindlimb.
