# Supplementary material for: The impacts of muscle-specific force-velocity properties on predictions of mouse muscle function during locomotion
Source: Front Bioeng Biotechnol. 2024 Jul 23;12:1436004. doi: 10.3389/fbioe.2024.1436004 (PMC11300213; doi:10.3389/fbioe.2024.1436004)
Supplement: Supplementary file 1 [file DataSheet1.zip › Supplementary Material.docx]

Electronic Supporting Information for:

**The impacts of muscle-specific force-velocity properties on predictions of mouse muscle function during locomotion**

James P Charles^1,*,#^, Roger W P Kissane^1*^ and Graham N Askew^2,#^

^1^ Department of Musculoskeletal & Ageing Science, University of Liverpool, The William Henry Duncan Building, 6 West Derby Street, Liverpool L7 8TX, UK

^2^ School of Biomedical Sciences, University of Leeds, Leeds LS2 9JT, UK

* Joint first author contribution

\# Correspondence to: j.charles@liverpool.ac.uk

# Sensitivity Analysis

Prior to the analysis described in the main text, an analysis was performed to determine the force-velocity curve parameters to which model and simulation outputs (muscle activation, force and power) were most sensitive in both extensor digitorum longus (EDL) and soleus (SOL). Here, several parameters (see Table S3) were altered individually by ±40% before running static optimisation simulations (12 in total) and calculating muscle power (see Methods). Root mean squared (RMS) errors were calculated to quantify the difference between each simulation and the default (no change- simulation 0) predictions.

The results (Table S4) highlight the high sensitivity of model predictions to each parameter, but particularly “Force value at the maximum normalised eccentric contraction velocity”, to which activation, force and power predictions were most sensitive.

# Cost of transport

To test the impact of changing individual muscle force-velocity properties on whole-body model outputs, the metabolic cost of transport (CoT) for the Generic and Extrapolated model simulations of Mouse 1 were calculated, along with individual muscle metabolic outputs and total “fast” and “slow” percentage contributions to overall CoT. Individual muscle metabolic output was predicted from the Generic and Extrapolated models via metabolic probes (Umberger et al., 2003), with CoT (Jkg^-1^m^-1^) calculated at each time step with the following formula:

$$CoT= \overline{\frac{P^{model}+ P^{non-Model}}{M_{b}*V}}$$

Where $P^{model}$ total modelled muscle power output, $P^{non-Model}$ is assumed power output from non-modelled structures (calculated from the mass of non-modelled segments multiplied by an assumed metabolic energy rate (1.2 Wkg^-1^) (Umberger et al., 2003)), $M_{b}$is body mass and $V$ is instantaneous velocity.

The results (Fig S6) show that while overall CoT didn’t change substantially between the Generic and Extrapolated models (Generic- 2.78 Jkg^-1^m^-1^; Extrapolated- 2.76 Jkg^-1^m^-1^), the percentage contribution from the fast muscle increased in the Extrapolated model (from 54.5% to 58.3%) and decreased in the slow muscles (from 5.83% to 5.18%).

Figure S1. Mucle-specific force-velocity curves, with raw data points, for mouse extensor digitoum longus (EDL) and soleus (SOL) muscles from Kissane and Askew (2024) which were input into the mouse musculoskeletal model in this study

Figure S2. Proportions of fibre types in mouse hindlimb muscles, as reported by Burkholder et al. (1994), and their separation into “slow”, “intermediate” and “fast” groups for the Extrapolated model. SOL- soleus, VI- vastus intermedius, GA- gracilis posterior, TA- tibialis anterior, GA- gracilis anterior, EHL- extensor hallucis longus, PLANT- plantaris, STa- semitendinosus anterior, STp- semitendinosus posterior, PL- peroneus longus, MG- medial gastrocnemius, EDL- extensor digitorum longus, FDL- flexor digitorum longus, RF- rectus femoris, VM- vastus medialis, TP- tibialis posterior, PT- peroneus tertius, PB- peroneus brevis, SM- semimembranosus, BF- biceps femoris, AL- adductor longus, AM- adductor magnus, LG- lateral gastronemius, VL- vastus lateralis.

Figure S3. Extensor digitorum longus (EDL) and soleus (SOL) activations and normalised fibre lengths as predicted by a trotting simulation and used as inputs into the *ex vivo* work loop.

Figure S4. Overview of the general study workflow. Muscle-specific force-velocity (F-V) curves for the extensor digitorum longus (EDL) and soleus (SOL) muscles were input into a musculoskeletal model and simulation of trotting within the mouse hindlimb, previously developed from descriptions of hindlimb anatomy and kinematics. This baseline model was scaled to match the body mass of 11 mice, creating 11 “real” (including muscle-specific F-V properties) and 11 “generic” (including generic F-V properties) models from which EDL and SOL power, work and function were predicted. These muscle-specific F-V were then extrapolated to other “fast” and “slow” muscles within the mouse hindlimb, creating 11 “extrapolated” models. Normalised nuscle fibre length changes and activations from these simulations were exported to an *ex vivo* preparation, from which EDL and SOL power, work and function were measured experimentally. These were compared to “extrapolated” model predictions in an attempt to validate the use of muscle-specific force-velocity data withinb musculoskeletal models.

Figure S5. Results of statistical parametric mapping (SPM) to test for significant differences between predicted extensor digitorum longus (EDL) and soleus (SOL) activation (a), force (b) and power (c) during one trotting stride with Real and Generic force-velocity properties. 1D-SPM maps (bottom panels of a, b and c) indicate regions of statistically significant differences between models, when SPM{t} lines exceed the critical threshold values denoted by the horizontal red dotted lines. Shaded regions within the SPM graphs correspond to the period within the gait cycle where the models were statistically significantly different from one another. Differences were mostly insignificant throughout the gait cycle, except for brief periods of significance before and after periods of activation and force generation in EDL in early swing (0-50% gait cycle) and early stance (50-100% gait cycle)

Figure S6. Metabolic output predictions between the Generic and Extrapolated models. Whole body cost of transport (a) predictions were similar between the models, however the % contribution of “fast” muscles increased in the Extrapolated model, while the contributions of “slow” muscles decreased.

Table S1. The number of mice which contributed to this study, via previously published work. Body masses and ages are reported as means ± standard deviations where appropriate.

| **Study** | **Contribution** | **Strain** | **Number** | **Body mass (g)** | **Age (days)** |
| --- | --- | --- | --- | --- | --- |
| Charles et al. (2016a) | Mouse hindlimb muscle architecture | C57BL/6 | 4 | 23.45±2.73 | 107.8±34.86 |
| Charles et al. (2016b) | Mouse hindlimb musculoskeletal model | C57BL/6 | 1 | 24.9 | 117 |
| Charles et al. (2018) | Mouse hindlimb kinematics & external forces | C57BL/6 | 5 | 18.7 ± 0.78 | 42 |
| Kissane and Askew (2024) | Muscle-specific force-velocity relationships | C57BL/6 | 21 | 25.06 ± 1.90 | n/a |
| This study | *Ex vivo* validation of model work and power predictions | C57BL/6 | 11 | 25.3 ± 2.1 | 70-84 |

Table S2. Individual body mass and muscle properties for each mouse used in the ex vivo work loop experiments.

| **Mouse** | **Body mass (g)** | **Muscle** | **Muscle mass (mg)** | **Optimal fibre length (m)** | **Maximum isometric force (N)** |
| --- | --- | --- | --- | --- | --- |
| **Charles et al (2016)** | 23.45 | EDL | 12.5 | 0.006 | 0.368 |
|  |  | SOL | 6.8 | 0.008 | 0.234 |
| **1** | 23.44 | EDL | 16.4 | 0.013 | 0.357 |
|  |  | SOL | 11 | 0.012 | 0.261 |
| **2** | 23.48 | EDL | 14.48 | 0.012 | 0.328 |
|  |  | SOL | 14.16 | 0.012 | 0.322 |
| **3** | 22.88 | EDL | 12.12 | 0.013 | 0.270 |
|  |  | SOL | n/a | n/a | n/a |
| **4** | 23.61 | EDL | 12.49 | 0.013 | 0.273 |
|  |  | SOL | 12.91 | 0.012 | 0.299 |
| **5** | 24.46 | EDL | 10.18 | 0.013 | 0.227 |
|  |  | SOL | 10.68 | 0.012 | 0.247 |
| **6** | 26.18 | EDL | 13.07 | 0.013 | 0.291 |
|  |  | SOL | 15.2 | 0.013 | 0.337 |
| **7** | 23.17 | EDL | 13.1 | 0.012 | 0.303 |
|  |  | SOL | n/a | n/a | n/a |
| **8** | 27.19 | EDL | n/a | n/a | n/a |
|  |  | SOL | 14.85 | 0.013 | 0.312 |
| **9** | 28.09 | EDL | 14.9 | 0.014 | 0.306 |
|  |  | SOL | n/a | n/a | n/a |
| **10** | 27.7 | EDL | n/a | n/a | n/a |
|  |  | SOL | 13.6 | 0.013 | 0.303 |
| **11** | 27.94 | EDL | 14.5 | 0.013 | 0.323 |
|  |  | SOL | 16 | 0.013 | 0.355 |
| Optimal fibre length in Mouse 1-11 was determined through a series of isometric twitches (0.2ms pulse width, supramaximal voltage) over 0.5mm increments. Optimal fibre length was the length that generated the peak force. | | | | | |

|  | **Parameter** | | | | | | | | | | | | | | | |
| --- | --- | --- | --- | --- | --- | --- | --- | --- | --- | --- | --- | --- | --- | --- | --- | --- |
|  | **EDL** | | | | | | | | **SOL** | | | | | | | |
| **Simulation** | **1** | **2** | **3** | **4** | **5** | **6** | **7** | **8** | **1** | **2** | **3** | **4** | **5** | **6** | **7** | **8** |
| **0 (no change)** | 14.13 | 0.008 | 0.032 | 0.125 | 0.25 | 5 | 1.4 | 0.6 | 7.01 | 0.017 | 0.063 | 0.125 | 0.25 | 5 | 1.4 | 0.6 |
| **1 (Fast 4 + 40%)** | 14.13 | 0.008 | 0.032 | ***0.175*** | 0.25 | 5 | 1.4 | 0.6 | 7.01 | 0.017 | 0.063 | 0.125 | 0.25 | 5 | 1.4 | 0.6 |
| **2 (Fast 5 + 40%)** | 14.13 | 0.008 | 0.032 | 0.125 | ***0.35*** | 5 | 1.4 | 0.6 | 7.01 | 0.017 | 0.063 | 0.125 | 0.25 | 5 | 1.4 | 0.6 |
| **3 (Fast 6 - 40%)** | 14.13 | 0.008 | 0.032 | 0.125 | 0.25 | ***3*** | 1.4 | 0.6 | 7.01 | 0.017 | 0.063 | 0.125 | 0.25 | 5 | 1.4 | 0.6 |
| **4 (Fast 7 - 40%)** | 14.13 | 0.008 | 0.032 | 0.125 | 0.25 | 5 | ***0.84*** | 0.6 | 7.01 | 0.017 | 0.063 | 0.125 | 0.25 | 5 | 1.4 | 0.6 |
| **5 (Fast 8 - 40%)** | 14.13 | 0.008 | 0.032 | 0.125 | 0.25 | 5 | 1.4 | ***0.36*** | 7.01 | 0.017 | 0.063 | 0.125 | 0.25 | 5 | 1.4 | 0.6 |
| **6 (Slow 4 - 40%)** | 14.13 | 0.008 | 0.032 | 0.125 | 0.25 | 5 | 1.4 | 0.6 | 7.01 | 0.017 | 0.063 | ***0.075*** | 0.25 | 5 | 1.4 | 0.6 |
| **7 (Slow 5 - 40%)** | 14.13 | 0.008 | 0.032 | 0.125 | 0.25 | 5 | 1.4 | 0.6 | 7.01 | 0.017 | 0.063 | 0.125 | ***0.15*** | 5 | 1.4 | 0.6 |
| **8 (Slow 6 + 40%)** | 14.13 | 0.008 | 0.032 | 0.125 | 0.25 | 5 | 1.4 | 0.6 | 7.01 | 0.017 | 0.063 | 0.125 | 0.25 | ***7*** | 1.4 | 0.6 |
| **9 (Slow 7 + 40%)** | 14.13 | 0.008 | 0.032 | 0.125 | 0.25 | 5 | 1.4 | 0.6 | 7.01 | 0.017 | 0.063 | 0.125 | 0.25 | 5 | ***1.96*** | 0.6 |
| **10 (Slow 8 + 40%)** | 14.13 | 0.008 | 0.032 | 0.125 | 0.25 | 5 | 1.4 | 0.6 | 7.01 | 0.017 | 0.063 | 0.125 | 0.25 | 5 | 1.4 | ***0.84*** |
| **11 (Fast all +/- 40%)** | 14.13 | 0.008 | 0.032 | ***0.175*** | ***0.35*** | ***3*** | ***0.84*** | ***0.36*** | 7.01 | 0.017 | 0.063 | 0.125 | 0.25 | 5 | 1.4 | 0.6 |
| **12 (Slow all +/- 40%)** | 14.13 | 0.008 | 0.032 | 0.125 | 0.25 | 5 | 1.4 | 0.6 | 7.01 | 0.017 | 0.063 | ***0.075*** | ***0.15*** | ***7*** | ***1.96*** | ***0.84*** |

Table S3. Force-velocity sensitivity analysis in extensor digitorum longus (EDL) and soleus (SOL). Various metrics were, in turn, changed by ±40% prior to running a static optimisation simulation. Bold and italicised numbers indicate values which were changed in a specific simulation. Parameter legend: 1- Maximum contraction velocity (ms^-1^), 2- Activation time constant, 3- Deactivation time constant , 4- Curve slope at maximum normalised concentric (shortening) velocity , 5- Curve slope just before reaching concentric slope at maximum normalised concentric (shortening) velocity, 6- Curve slope at isometric (normalised velocity of 0) , 7- Force value at the maximum normalised eccentric contraction velocity, 8- Concentric curviness.

Table S4. Sensitivity analysis results, with root mean squared (RMS) errors expressed as percentages. See Table S2 for details on each simulation

|  | **EDL** | | |  | **SOL** | | |
| --- | --- | --- | --- | --- | --- | --- | --- |
|  | ***RMSE %*** | | |  | ***RMSE %*** | | |
| ***Simulation*** | ***Activation*** | ***Force*** | ***Power*** | ***Simulation*** | ***Activation*** | ***Force*** | ***Power*** |
| **1** | 0.037 | 0.062 | 0.057 | **6** | 0.024 | 0.044 | 0.032 |
| **2** | 1.239 | 2.153 | 1.535 | **7** | 1.459 | 0.559 | 1.758 |
| **3** | 3.328 | 6.619 | 4.632 | **8** | 0.648 | 0.419 | 0.805 |
| **4** | 8.236 | 14.182 | 10.831 | **9** | 10.140 | 27.334 | 90.000 |
| **5** | 3.968 | 7.629 | 5.483 | **10** | 2.375 | 1.295 | 2.466 |
| **11** | 9.320 | 21.256 | 18.989 | **12** | 5.783 | 26.968 | 97.038 |

**References**

BURKHOLDER, T. J., FINGADO, B., BARON, S. & LIEBER, R. L. 1994. Relationship between muscle fiber types and sizes and muscle architectural properties in the mouse hindlimb. *J Morphol,* 221**,** 177-90.

CHARLES, J. P., CAPPELLARI, O. & HUTCHINSON, J. R. 2018. A Dynamic Simulation of Musculoskeletal Function in the Mouse Hindlimb During Trotting Locomotion. *Frontiers in Bioengineering and Biotechnology,* 6.

CHARLES, J. P., CAPPELLARI, O., SPENCE, A. J., HUTCHINSON, J. R. & WELLS, D. J. 2016a. Musculoskeletal Geometry, Muscle Architecture and Functional Specialisations of the Mouse Hindlimb. *PLoS One,* 11**,** e0147669.

CHARLES, J. P., CAPPELLARI, O., SPENCE, A. J., WELLS, D. J. & HUTCHINSON, J. R. 2016b. Muscle moment arms and sensitivity analysis of a mouse hindlimb musculoskeletal model. *J Anat,* 229**,** 514-35.

KISSANE, R. W. P. & ASKEW, G. N. 2024. Conserved mammalian muscle mechanics during eccentric contractions. *J Physiol,* 602**,** 1105-1126.

UMBERGER, B. R., GERRITSEN, K. G. & MARTIN, P. E. 2003. A model of human muscle energy expenditure. *Comput Methods Biomech Biomed Engin,* 6**,** 99-111.
